# Improving maternal well-being: a matrescence education pilot study for new mothers

**DOI:** 10.1186/s40748-025-00203-0

**Published:** 2025-07-02

**Authors:** Victoria Trinko, Julia Sarewitz, Aurelie Athan

**Affiliations:** Teachers College, Columbia University, New York, US

**Keywords:** Matrescence, Maternal Well-Being, Transition to Motherhood, Positive Psychology, Reproductive Health Literacy, Maternal Mental Health, Perinatal Distress

## Abstract

**Background:**

The transition to motherhood, known as matrescence and comparable to adolescence, involves significant changes across multiple life domains, impacting maternal identity and increasing the risk of psychopathology. Conventional maternal mental health interventions often emphasize crisis management over proactive resilience building. Psychoeducational programs designed to empower and support positive adaptation may offer a beneficial preventative approach. This pilot study evaluates the acceptability, relevance, and effectiveness of a matrescence-informed maternal health education program developed to enhance new mothers’ understanding of the complex emotional and social challenges associated with the transition to motherhood.

**Methods:**

This study evaluated a six-week, matrescence-informed maternal health education program delivered via Zoom. Eighteen participants (*n* = 18) attended weekly 75-minute sessions that included lectures, experiential exercises, and group discussions. Pre- and post-intervention surveys were administered to assess mindfulness, self-compassion, perceived stress, personal growth, and psychological well-being. Quantitative data were analyzed using paired t-tests to compare pre- and post-intervention scores, and qualitative responses were analyzed using thematic analysis to capture participant perceptions of the program’s relevance and impact.

**Results:**

The pilot program demonstrated improvements in select psychological measures. While overall mindfulness scores did not change, increases were noted in the subscales of observing, non-judgment, and non-reactivity. Self-compassion scores increased, and participants reported gains in areas such as personal strength, relationships, and spiritual development. Perceived stress and psychological well-being remained unchanged, though environmental mastery showed improvement. Qualitative responses described challenges related to physical and emotional challenges as well as identity shifts. Participants assessed the program to be relevant and useful in addressing the challenges of motherhood and in enhancing their awareness of the concept of matrescence.

**Conclusions:**

Positive participant feedback suggests that matrescence-informed education may offer benefits to new mothers' understanding of their developmental transition and should be disseminated more widely to improve their awareness and literacy. The small sample size highlights the need for larger-scale studies, including randomized controlled trials and longitudinal follow-ups, to evaluate the program's potential in mitigating the risk of poor maternal mental health outcomes. Efforts should be made to reduce barriers to matrescence education and make it affordable and accessible to all.

## Introduction

Becoming a mother is a major life transition involving dramatic changes to a woman’s brain, body, relationships, societal roles, and identity. This shift necessitates significant psychological and social adjustments, which can challenge mothers’ self-perception and confidence, and heighten their vulnerability to mental health issues such as postpartum depression, anxiety, and even psychosis [26, 88]. Hormonal changes, sleep deprivation, and the stress of caregiving often exacerbate these challenges, especially in the absence of adequate support. Despite a 13–20% prevalence of depressive symptoms among new mothers, only 20% are screened for postpartum depression [18, 45]. Maternal mental illness is the leading cause of pregnancy-related deaths in the U.S., responsible for 1 in 4 maternal deaths [106]. Poor maternal health not only imposes substantial economic burdens on society but also impacts child development, and intensifies social stigma and isolation for mothers. This underscores the urgent need for preventive education and early intervention to safeguard maternal well-being before critical issues emerge [21, 100]. Reports from the American College of Obstetrics and Gynecology (ACOG), World Health Organization (WHO), and Centers for Disease Control (CDC have reflected this, advocating for more proactive guidance and demedicalized, multidisciplinary, and community-based models of care to address the escalating maternal health crisis and improve health outcomes [1, 20, 111].

In the past decade, increased recognition and education around perinatal mood and anxiety disorders (PMADs) have significantly improved public and professional understanding and encouraged more compassionate, mother-centered mental healthcare. Recently, advocacy efforts have shifted from solely focusing on reducing psychopathology to promoting resilience and overall well-being in mothers. Initiatives from World Maternal Mental Health Awareness Day [108] and the WHO have emphasized reframing maternal mental health as more than the absence of illness but as a state of flourishing [107] and have championed for anticipating the supports needed for mothers by helping them identify their resources and personal capabilities earlier in the perinatal period. This shift towards a strengths-based, holistic approach to maternal well-being should inspire future healthcare designs.

One such innovation could be integrating the concept of matrescence—the developmental transition to motherhood—into public awareness campaigns, health education programs, and training for care providers (see detailed definition of the concept in the following section). This approach could fill systemic gaps, provide continuous, interlinked support from pregnancy through early motherhood, and meet the comprehensive needs of mothers during this vulnerable period [21, 28, 34, 55, 68, 100].

### Matrescence Like Adolescence: A Developmental Perspective

Matrescence, a term introduced by medical anthropologist Dana Raphael in 1973, defines the transition to motherhood as a developmental rite of passage, much like adolescence, with biological and social implications [82]. Athan and Reel [3] later expanded this concept to encompass the more extensive biopsychosocial and ideological changes that individuals undergo during this period. They noted that while these changes can lead to emotional upheaval, they can also result in positive outcomes such as increased self-awareness, crystallized values, and psychological maturation. Unlike traditional biomedical views that might inadvertently pathologize stress responses during motherhood as conferring risk only, a developmental perspective views them also as opportunities for growth—much like the transition into adulthood for adolescents if they are appropriately supported.

What makes matrescence distinct from adolescence is that its length varies among individuals, it may reoccur with each new child, and may have an acute stage followed by a more complex, and longer process with no definitive end (e.g., grandmothering). It has been suggested that integrating developmental psychology terms like “emerging motherhood” (infancy to school age), “middle motherhood” (adolescence), and “late motherhood” (adulthood) could enhance our understanding of the evolving stages within this lifelong transition. Despite its potential utility, matrescence has remained largely overlooked in mainstream psychological theories and allied perinatal health disciplines since it was first introduced. More recently, matrescence has begun to be utilized as an explanatory framework. For example, developmental neuroscience studies have revealed that a mother’s brain undergoes considerable neuroplastic changes during this period, enhancing areas responsible for bonding, social sensitivity, memory, attention, executive function, and sleep [10, 19, 44, 49, 75, 76]. Psychologically, new mothers have been found to navigate identity diffusion, increased acceptance and commitment of their role requiring ego resilience such as tolerance of ambiguity, cognitive flexibility, emotional regulation, self-related processing, and social cognition [11, 23, 48, 65]. Ideologically, the shift into motherhood can involve feminist awakenings to unfair gender role expectations or difficulties in balancing work and family life that can negatively impact labor retention [29, 53, 84]. Maternal ecodistress is also a growing area of research, demonstrating how environmental issues like climate change and natural disasters shape maternal mental health [14, 25, 85]. Additionally, studies on maternal morality and spirituality suggest that matrescence fosters mothers to embrace tenants common to the world’s wisdom traditions such as non-violence, compassion, mindfulness, and faith [4, 58].

**Fig. 1:**
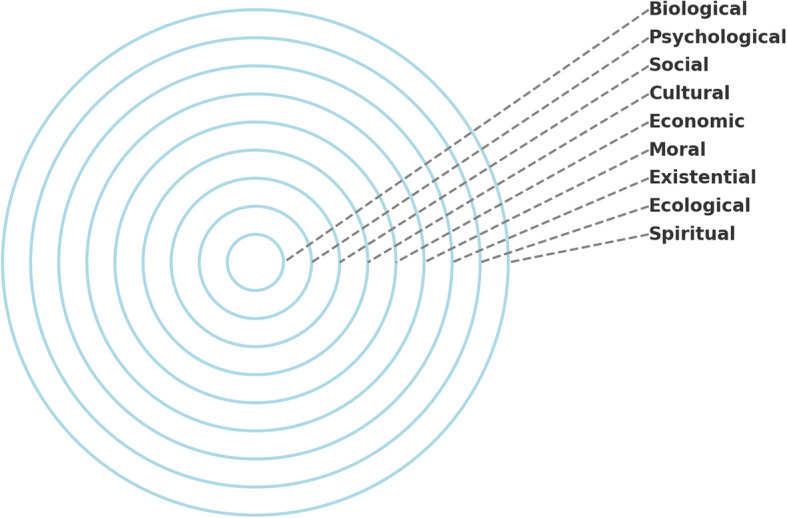
Developmental Domains of Matrescence. Note: Order of domains may undergo reorganization with more study

The above findings contribute to a more comprehensive understanding of matrescence, and suggest that the transformations that occur during preconception, pregnancy, birth, surrogacy, adoption, and early parenting can affect every domain of human life experience, above and beyond those initially identified by Raphael. In response, a more updated definition was provided by Athan [2] to include it as a holistic phenomenon, or a, “…lifespan, developmental transformation that is biological, neurological, psychological, social, cultural, economic, political, moral, existential, ecological, and spiritual in nature”. This expanded conceptualization is essential for facilitating a thorough understanding of motherhood that may more aptly explain the decline in maternal well-being observed today (See Fig. 1.)

### Reframing Maternal Distress in Matrescence

A recent report by Murthy [67], *Parents Under Pressure: The U.S. Surgeon General’s Advisory on the Mental Health and Well-being of Parents* was released by the U.S. Department of Health and Human Services. This advisory highlights the significant stress many parents and caregivers face, with 41% reporting that they are "so stressed they cannot function" most days, and nearly 50% stating that their stress feels overwhelming most of the time [67]. This "silent majority" of mothers experiences a wide range of symptoms referred to generically as "maternal distress" [22, 33]. While the more extreme ends of this spectrum include diagnosable mental health conditions (20%) on the one side and potentially perinatal flourishing (20%) on the other, most mothers will fall into the "messy middle" marked by feelings of ambivalence such as joy and satisfaction, alongside apprehension and exhaustion. This highlights the complexity of matrescence, which is characterized by both psychological instability *and* growth. Mezirow’s [61] theory of transformative learning offers a valuable framework for understanding how adults reshape their interpretations of the world by critically reflecting on their experiences (Taylor, 2017). Central to this theory is the notion that major life transitions can prompt a revision of one’s frame of reference—those deep-seated assumptions and expectations that shape beliefs and behaviors. By applying this lens to matrescence, maternal distress can be reimagined not only as a pathological condition, but as a “disorienting dilemma,” where mothers challenge previous self-understandings and construct new worldviews more aligned with their emerging realities [62, 63]. Rather than stigmatizing the non-clinical range of stress responses that mothers exhibit during this period, reframing them as part of a normative developmental process may improve our understanding of maternal adaptation and offer insights into how best to help those who struggle.

Ben-Ari, Shlomo, Findler, and Sharon [12] also liken this process to *post-traumatic growth*, where loss of control, emotional distress, and behavioral confusion are present but ultimately lead to positive adaptation. Mothers must reconcile their former self-concepts with the demands of caregiving, often grieving the loss of previous lifestyles, personal freedoms, and career aspirations while learning to embrace the more rewarding aspects of motherhood. Painful physical experiences, such as difficult births or breastfeeding complications, can prompt existential questioning, revisiting value systems, or bringing repressed traumas to the surface. The pressures to excel in both parenting and the workplace can overwhelm mothers, eroding their efforts to maintain pre-motherhood social connections and leading to unexpected isolation. Much like the “growing pains” of adolescence, matrescence offers significant opportunities to reassess roles, responsibilities, and identities, allowing mothers to establish different priorities and uncover personal strengths. Unfortunately, the lack of quality maternal health education and preparation for mothers may contribute to their poor psychological coping, placing them at higher risk for falling ill rather than fostering their resilience. Resilience is defined as the ability to adapt positively in the face of adversity, and may be a key dimension to target preventively if we are to help mothers bounce back from the stressors of parenthood and to thrive rather than merely survive future mental health crises. Research consistently shows that resilience is strongly correlated with overall well-being, aiding in stress management, emotional stability, and recovery from trauma [16, 59, 94].

Mothers need evidence-based guidance to navigate this transition, yet many programs prioritize the child's needs over the mother's, and are often mediated by providers untrained in maternal mental health [21, 100]. Surveys show that only 41% of mothers feel adequately informed post-childbirth and 47% feel prepared for self-care upon leaving the hospital [6]. Additionally, 79% of mothers feel invisible and 95% feel unacknowledged and unappreciated [77]. Healthcare practices typically focus on newborns, with first post-birth appointments often with pediatricians rather than OBGYNs [39, 101]. A visit with an OBGYN usually does not occur until four to six weeks postpartum—primarily for physical recovery, contraception counseling, and ideally a mental health screening—leaving a critical gap in care during the earliest and often most vulnerable weeks after childbirth. Cultural norms emphasizing maternal self-sacrifice and reliance on “maternal instinct” further disincentivize mothers from seeking out more education or advocating for their needs [38]. Additionally, the lack of paid parental leave and affordable childcare options in the U.S. further exacerbate these issues, placing even greater strain on mothers as brought to light during the COVID-19 pandemic [31, 42, 50, 56, 80, 92].

### Enhancing Maternal Health Literacy

Despite the critical role of education in nurturing and empowering mothers during the perinatal period, a substantial gap persists between the information mothers seek and what they actually receive—an “unmet need” in maternal care [40]. The frequent lament among new mothers, “Why didn’t anyone tell me?” underscores the inadequacy of basic education, which can negatively impact maternal outcomes by limiting awareness and informed decision-making [27, 89, 91]. Improved health literacy, defined as the ability to access, comprehend, and apply relevant health information, is essential for equipping women with the knowledge needed to navigate the complex transition to motherhood [73, 83]. Low health literacy, in particular, has been shown to significantly increase the risk of poor outcomes, further emphasizing the need for comprehensive maternal education [15].

Historically, programs such as childbirth education, primarily focus on the physiological and behavioral aspects of pregnancy, birth, and infant feeding. There exists a lack of programs that address the full spectrum of motherhood, particularly its lifespan, developmental, and psychosocial aspects [41, 57, 93, 95]. More holistic approaches typically more available to adolescents during their transition to adulthood offers a useful example for how to design such educational programs. For example, Positive Youth Development (PYD) programming offers emotional support, practical guidance, and skill development to adolescents to help them think critically and flexibly adapt to their emerging responsibilities and identity changes. This proactive approach normalizes the stressful road to adulthood by nurturing the inherent strengths of youth and supporting them to improve their social and emotional competencies before and prevent future negative outcomes [47, 87, 113].

Similarly, matrescence education aims to bolster the self-righting adaptive capacities of mothers and to prepare them with the positive coping skills necessary to endure the chronic issues of motherhood. By providing a combination of evidence-based knowledge, practical skill-building tools, and peer social support, matrescence education can help mothers become competent, confident, and nurturing caregivers *and receivers*. Table 1. describes how matrescence education can enhance maternal well-being by highlighting its key areas of impact to ensure mothers receive the holistic care they require.

**Table 1:** Key Areas of Impact when Implementing Matrescence-Informed Education

Areas of Impact	Summary
Normalization & Knowledge Enhancement	Destigmatize and validate maternal distress as common, manageable, and deserving of support. Enhance mothers’ maternal health literacy by introducing the term "matrescence" and explain the normative changes expected during the transition to motherhood
Transformative Learning & Growth Mindset	Equip mothers with the tools to critically reflect on and reshape their belief systems, promoting psychological flexibility and openness to experience. Encourage them to view challenges as opportunities for learning and further development of their adaptive capacities
Resilience Building & Stress Management	Incorporate positive coping skills training and resilience-building to reduce the risk of developing more severe mental health issues. Techniques such as mindfulness and self-compassion can enhance emotional regulation and help manage stress
Peer Support & Community Networking	Provide mothers with guidance on how to leverage community support systems and opportunities for peer learning through shared experiences to alleviate isolation, loneliness, and act as a buffer against feelings of inadequacy and negative social comparison
Resource Accessibility & Visibility	Improve mothers' awareness of and access to maternal mental health resources, by distributing information and referral sources, thereby reducing barriers to essential services and encouraging mothers to locate and seek out help through formal and informal when needed

#### Normalization and Knowledge Enhancement

As previously discussed, effective matrescence education starts with enhancing maternal health literacy. A key component of this education involves explaining the concept of matrescence and helping them apply it to discern between clinical and non-clinical reactions to the multitude of changes they are experiencing. This increased awareness enables mothers to differentiate between normal developmental stress and more serious symptoms in terms of degree and frequency that may require professional intervention. Normalizing maternal distress as a common aspect of the transition to motherhood that can be successfully managed *when properly supported* can also reduce stigma and increase the self-esteem of mothers. This more comprehensive education, alongside more conventional pregnancy and childbirth preparation classes, will hopefully lay the groundwork for mothers to engage in a love of lifelong learning for reproductive issues (e.g., menopause). It may also build longer-term relationships with care providers who can be seen as trusted allies who will validate rather than dismiss their concern that there is something uniquely wrong with them [34, 70, 100, 105].

#### Transformative Learning and Growth Mindset

Emphasizing continuing adult learning, its intrinsic value, and potential for personal growth throughout the lifespan can strengthen mothers’ motivation to deepen their self-awareness and knowledge of the evolving scientific literature on reproductive health. This approach should move beyond mere memorization of facts from top-down authorities, which can undermine agency and self-efficacy. Instead, matrescence-informed education should incorporate transformative learning principles that encourage mothers to critically examine their beliefs and assumptions through experiential exercises and introspective activities. By viewing their disorientation and discomfort as an opportunity for self-discovery, mothers may engage in more creative problem-solving which may in turn foster a more optimistic outlook. Motivating mothers to actively derive meaning from their experiences, reprioritize their goals, and seek greater clarity are the hallmarks of a growth mindset [13].

#### Resilience Building and Stress Management

Building resilience is a dynamic process that involves strengthening healthy responses while learning to prepare for adverse events. This can be achieved by leveraging existing character strengths to effectively cope with the demands of raising children [37, 110]. Helping mothers learn techniques from contemplative traditions and positive psychology such as mindfulness meditation, gratitude journaling, and learned hopefulness can help reduce stress, negative self-talk, hypervigilance, and perfectionism [17, 32, 43, 54, 78, 112]. These practices counter maladaptive attitudes correlated with poor maternal mental health by promoting non-judgmental awareness and openness to experience instead [24, 46, 104]. Feminist approaches such as deconstructing societal ideals and creating more realistic personal expectations can also be utilized to promote greater self-esteem and gender equity in the home associated with improved parenting satisfaction [35, 66, 74].

#### Peer Support and Community Networking

Research shows that resilience is not just as an individual trait but one that can be strengthened by supportive contexts [103]. Motherhood often involves a reorganization of peer, partnership, and familial relationships which can lead to feelings of loneliness and isolation [51, 69]. The round-the-clock caring for a newborn can further reduce help-seeking behaviors [26, 52]. Educational programs that emphasize community building and growing social networks provide mothers with practical, informational, and emotional assistance. Peer learning through shared experiences should be the mainstay of such programs to counteract social-comparison and feelings of inadequacy. Facilitating a sense of belonging and connectedness with like-minded others has been shown to contribute to increased self-competence and greater satisfaction in the maternal role [30, 64, 72].

#### Resource Accessibility and Visibility

Maternal health educational programs should explicitly provide informational resources to mothers such as reading materials, counseling services, support groups, and local healthcare providers. Increasing accessibility can help them navigate the complex maternal health landscape and reduce help-avoidance behaviors [27, 91]. Additionally, programs should guide mothers to identify, enhance the visibility of, and evaluate the effectiveness of their existing information, emotional, and practical resources in meeting their needs. Encouraging mothers to critically evaluate gaps in their self-care may empower them to request improvements through grassroots initiatives or informal channels. Facilitators can further promote local services, ensure clearer communication about available resources, and support mothers in sharing and advocating for services within their communities when needed.

## Methodology

### Study design

The original draft of the workshop was developed by two non-licensed, Master’s-level psychology students as part of a practicum course. This early version served as the foundation for continued refinement in collaboration with a PhD-level faculty member and licensed psychologist, who guided its theoretical alignment with matrescence and its structural adaptation to support the study’s research questions and pre–post evaluation design. A final, revised version was piloted and tested in the present study. Student facilitators led weekly sessions under close faculty supervision to ensure fidelity to the research protocol and adherence to ethical standards. The study received approval from the Institutional Review Board of the university. All participants provided informed consent after being thoroughly briefed on the study's objectives, the voluntary nature of participation, potential risks, and the measures taken to ensure confidentiality. Data collection was anonymized and securely stored to protect participant privacy.

The program was delivered through an institutionally-sanctioned online video conference platform, Zoom. Each week consisted of a 75-minutes co-facilitated group session, incorporating a blend of didactic lectures, experiential skill-building exercises, and live group discussions. Participants were informed that the series would center on the emerging concept of matrescence, viewing it as an opportunity for self-exploration and building their resilience. Unlike conventional perinatal education, which typically focuses on childbirth preparation, parenting techniques, or PMADs, this program emphasized personal growth and self-awareness for the sake of the mothers themselves.

Key components of the program included lectures on the domains of matrescence and resilience-building experiential exercises that integrated principles from Developmental Psychology, Positive Psychology, and Transformative Learning Theory. The program also focused on normalizing challenges through group discussions and peer learning to alleviate isolation and foster a sense of belonging. Resources were also distributed for relevant maternal mental health organizations. Between sessions, participants engaged in self-reflective journaling and homework assignments to integrate concepts explored each session and apply them to their daily demands. The themes, key activities, and objectives explored in each session have been outlined below (see Table 2.).

**Table 2:** Session Themes, Key Activities, and Objectives

Session	Theme	Key Activities	Objectives
1	Introduction to Matrescence	Overview of matrescence, maternal autobiographical exercise	Situate personal experience within concepts of human developmental psychology, initiate self-reflection
2	Domain Changes of Matrescence	Overview of domain changes, self-compassion practice	Elaborate challenges faced in each domain, enhance self-understanding
3	Resilience Skill Building	Overview of skill-building, mindfulness practice	Strengthen personal practices, promote emotional self-regulation
4	Psychology of Transformation	Overview of transformative learning principles, character strengths exercise	Reframe disorientation as growth-producing, identify internal resources
5	Community Building	Overview of social support, support network exercise	Expand personal networks, identify external resources
6	Resource Amplification	Overview of themes, toolkit exercise	Develop personal resources, integrate learning in daily life

The objectives of this pilot study were to:

1. Evaluate the Concept of Matrescence: Determine the concept of matrescence’s relevance and utility for participants navigating the transition to motherhood.
2. Measure Program Impact on Maternal Well-Being: Examine the program’s effect on positive psychology assessments chosen for this study to gauge improvements in overall maternal well-being: mindfulness, self-compassion, personal growth, psychological well-being, and perceived stress.
3. Evaluate Program Acceptability: Determine participants' perceptions of the program’s acceptability for addressing the challenges encountered during their matrescence.

Participants were recruited through a combination of printed flyers, email announcements, word-of-mouth, and social media posts. Inclusion criteria was as follows: “Our inclusion criteria is any self-identifying mother with children 0–4 years of age in the U.S. Because matrescence, the developmental transition to motherhood, is centered on the mothers’ experience, we have selected to focus solely on mothers who have recently gone through the transition and who can likely recall their experience for the purposes of our research. We will accept anyone who self-identifies as a mother, i.e. all those who make caregiving the next generation a central part of their lives, including all the ways people come to that work.”

Upon expressing interest, potential participants were screened using an online questionnaire to ensure they met the inclusion criteria. Those who qualified completed a baseline pre-test survey and a post-test survey after completion of the 6-week program which included quantitative measures, open-ended qualitative questions and an overall course evaluation. The study received interest from 28 people and accepted 20 participants, with 18 completing the program due to attrition. Reasons given for attrition included difficulty finding time in their schedule or lack of childcare. Participants were divided into two groups based on availability (Group 1: *n* = 11; Group 2: *n* = 7). The attendance rate was documented with 27.78% of participants with low attendance, 16.67% of participants with moderate attendance, and 55.56% of participants with high attendance. Participants were not compensated for their time and participation was completely voluntary.

### Demographic characteristics

The final study sample consisted of 18 mothers across the perinatal window and beyond. This range allowed the program to capture a wide spectrum of experiences related to matrescence, from the anticipation of motherhood to mothering infants and toddlers. Of the participants, 4 were pregnant, with 2 experiencing pregnancy for the first time (primiparous). The remaining 14 participants were not pregnant at the time of the study and were mothers to children ranging from infants to four years old. The majority of participants were highly educated, with 50% holding advanced degrees (master's or Ph.D.) and married (83.3%). In our initial pre-assessments, socioeconomic status (SES) was not explicitly measured; however, subsequent intake data, including access to healthcare, mental healthcare, higher education levels, may indicate that all participants fall within middle to high SES categories.

### Measures

Five positive psychology scales were employed to evaluate changes in participants’ self-report of perceived well-being. Measures of mindfulness, self-compassion, post-traumatic growth, perceived stress, and psychological well-being were selected due to their established roles as key indicators in resilience-building, psychological well-being enhancement, or the promotion of stress reduction [5, 99, 102].

#### The Five Facet Mindfulness Questionnaire

The Five Facet Mindfulness Questionnaire (FFMQ), developed by Baer et al. in 2006 [9], is a widely used self-report questionnaire designed to assess various components of mindfulness. It aims to measure an individual's ability to be present and attentive to their experiences in a non-judgmental and accepting manner. The FFMQ consists of 39 items, with seven or eight questions for each facet. Participants rate the extent to which each statement applies to them on a five-point scale ranging from 1 ("Never or very rarely true") to 5 ("Very often or always true"). Total scores on the FFMQ range from 39 to 195, with higher scores indicating higher levels of mindfulness. Across various studies, the internal consistency of the FFMQ has been found to be high, with Cronbach's alpha coefficients typically ranging from 0.80 to 0.90 for the total scale. For individual facets, Cronbach's alpha coefficients generally range from 0.70 to 0.90 [7, 60, 90].

#### Self-Compassion Scale Short Form

The Self-Compassion Scale Short Form (SCS-SF) is a brief self-report questionnaire developed by Raes et al. in [81]. It was developed as a short form of Neff’s Self Compassion Scale [71], which aims to measure self-compassion, which involves being kind and understanding toward oneself in instances of perceived inadequacy or failure. The SCS-SF consists of 12 items, with two items for each component. Participants rate the extent to which each statement applies to them on a five-point scale ranging from 1 ("Almost Never") to 5 ("Almost Always"). Total scores on the SCS-SF range from 1–5, with higher scores indicating higher levels of self-compassion. Scores of the short form of this scale are only intended to be used for comparative purposes. The Cronbach's alpha coefficient for the SCS-SF typically falls within a range similar to that of the original Self-Compassion Scale (SCS), which is around 0.80 to 0.90. This indicates strong internal consistency and reliability for the short form version of the scale [81].

#### Posttraumatic Growth Inventory

The Posttraumatic Growth Inventory (PTGI) is a 21-item self-report questionnaire designed to measure positive psychological changes that occur as a result of experiencing trauma or adversity. Developed by Tedeschi and Calhoun [98], the PTGI assesses five domains of posttraumatic growth. Participants are asked to rate the extent to which each item reflects their experiences on a six-point scale ranging from 0 ("I did not experience this change as a result of my crisis") to 5 ("I experienced this change to a very great degree as a result of my crisis"). Higher total scores on the PTGI suggest greater posttraumatic growth, with a total score range from 0 to 105. The Cronbach's alpha coefficient for the Posttraumatic Growth Inventory (PTGI) typically falls within a range of 0.90 to 0.95, indicating very high internal consistency and reliability [79, 97, 98].

#### Perceived Stress Scale

The Perceived Stress Scale (PSS) is a widely used psychological instrument for measuring the perception of stress in individuals. Developed by Cohen, Kamarck, and Mermelstein in 1983, the scale aims to assess how unpredictable, uncontrollable, and overloaded individuals find their lives. The PSS is designed to capture the degree to which situations in one's life are appraised as stressful, rather than measuring specific stressors. The scale consists of several questions that ask about feelings and thoughts during the last month. Participants are asked to rate how often they felt a certain way on a 5-point Likert scale, ranging from 0 (Never) to 4 (Very often). The total score is calculated by summing the responses to all items, with higher scores indicating higher perceived stress levels. The PSS has been shown to have good internal consistency, typically with Cronbach's alpha coefficients ranging from 0.70 to 0.90 [109].

#### Psychological Well-Being

The Psychological Well-Being Scale (PWB), developed by Ryff in 1989, is a self-report questionnaire designed to assess six dimensions of psychological well-being. The PWB consists of 84 items, with 14 items for each dimension. Participants rate each item on a six-point scale ranging from 1 ("Strongly Disagree") to 6 ("Strongly Agree"). Higher total scores on the PWBS indicate higher levels of psychological well-being across its dimensions. Total scores can range from 84 to 504. The internal consistency of the PWB Scale is typically high, with Cronbach's alpha coefficients ranging from 0.80 to 0.90 across different studies and populations [86].

### Qualitative Questions

Open-ended questions were administered during both the pre- and post-assessments to gain a comprehensive understanding of each participant's unique experience of matrescence. Pre-assessment questions focused on capturing a picture of participants' early experiences of new motherhood prior to the intervention. Examples of pre-assessment questions include: Before your transition to motherhood, how did you feel about becoming a mother?; How easy or difficult was pregnancy for you (if you were the birthing person)? Please consider any emotional, physical, or relational aspects of your experience.; How easy or difficult is new motherhood for you? Please consider any emotional, physical, or relational aspects of your experience; and Did you take any courses or seek out resources to prepare for pregnancy, childbirth, or new motherhood? If so, what courses or resources? Were they helpful to you?

Post-assessment questions aimed to evaluate two core objectives of the study: the concept of matrescence and the acceptability of the program. Examples of the questions asked for the purpose of evaluating the concept of matrescence include: Has learning about matrescence changed the way you understand or perceive your transition to becoming a mother?; Has reflecting on your transition to becoming a mother through the lens of matrescence helped you reframe your current experience in new motherhood?; and Do you find the concept of matrescence to be applicable to your experience of becoming a mother? Questions intended to measure the acceptability of the program include: Was the information presented in the workshop was useful, practical and applicable?; What part of the workshop did you find most and/or least valuable?; and To what extent has the workshop equipped or prepared you to handle motherhood more effectively? Testimonial questions were also asked but not included in the qualitative analysis.

### Data Analysis

Quantitative data was statistically analyzed using paired-sample t-tests to compare pre- and post-test scores, allowing assessment of changes over time and the program’s longitudinal benefits. Statistical significance was set at *p* < 0.05, with p-values < 0.01 and < 0.001 indicating high and extremely high significance, respectively. Out of the total participants, 18 completed the pre-test survey, and 14 completed the post-test survey, though one participant skipped the PTGI in both the pre and post assessments. The analysis was restricted to the 14 participants who completed both surveys. Qualitative data was analyzed through thematic analysis to identify and systematically analyze patterns, themes, and meanings within participants' responses to open-ended questions. This approach helped categorize recurring themes, offering contextual understanding of the quantitative results. The integration of both data types provided a comprehensive view of the program's effectiveness, allowing for a nuanced interpretation of how it influenced participants' well-being and experiences. To ensure confidentiality and traceability, participants were assigned unique identifiers (e.g., #1, #2), with any quotes referenced in the analysis labeled accordingly.

## Results

### Quantitative Results

The quantitative analyses revealed varying degrees of impact across the five measures detailed below and in the table provided (see Tables 3, 4 and 5). These findings reflect positive shifts in many psychological constructs related to well-being and resilience, highlighting an upward trajectory in mental health and adaptive coping mechanisms among participants.

**Table 3:** Participant Demographics

Variables	Participants (*n* = 18)
Age (years)	
Mean	36.1
Min–Max	29–41
Median	36
Gender (%)	
Cisgender Woman	94
Non-Binary	6
Identify as “mother”	100
Location (%)	
US South	17
US Northeast	66
US Midwest	0
US West	17
Education Level (%) Highest Degree Attained	
Undergraduate	50
Masters	28
PhD	22
Marital Status (%)	
Married	83
Single	6
Domestic Partnership	11
Sexual Orientation (%)	
Heterosexual	67
Bisexual	6
Queer	6
Asexual	6
Questioning or Unsure	6
Race/Ethnicity (%) Caucasian	67
Multi-racial	18
Hispanic/Latina (Black)	5
Hispanic/Latina (White)	5
Asian American/Pacific Islander	5
Religious Affiliation (%)	
Religious: Christian	11
Religious: Jewish	6
Religious: Buddhist	6
Religious: Other	6
Religious: Interfaith	16
Atheist/Agnostic	22
Spiritual, Not Religious	33
Employment Status (%)	
Full Time Work	56
Part-Time Work	11
Full-Time Parent	17
Unemployed	5
Full-Time Student	5
Contractor	6
Parity (%)	
1 Child	44
2 Children	33
Expecting + 1 Child	11
Expecting + 0 Children	11
Age of Oldest Child (%)	
Pregnant	11
0–12 Months	28
12–24 Months	28
Between 2- 3 Years	22
Between 3–4 Years	11

**Table 4:** Measures, Subscales and Sample Questions

Scales with Subscales	Description and Sample Questions
Five Facet Mindfulness Questionnaire (FFMQ)	
Observing	The ability to notice and attend to internal and external experiences, such as sensations, thoughts, and emotions: "I notice the smells and aromas of things"
Describing	The ability to put internal experiences into words and describe them accurately: "I'm good at finding words to describe my feelings."
Acting with Awareness	The ability to engage fully in one's activities and be present in the moment: "I find it difficult to stay focused on what's happening in the present.”
Non-Judging of Inner Experience	The tendency to observe thoughts and feelings without evaluating them as good or bad: "I tell myself I shouldn't be feeling the way I'm feeling.”
Non-Reactivity to Inner Experience	The ability to allow thoughts and feelings to come and go without becoming overly attached to or reactive to them: "When I have distressing thoughts or images, I am able to just notice them without reacting.”
Self-Compassion Scale (SCS-SF)	
Self-Kindness	Being warm and understanding toward oneself rather than harshly self-critical: “I try to be understanding and patient towards those aspects of my personality I don’t like.”
Self-Judgment	Avoiding harsh self-criticism and judgment (R): “I’m intolerant and impatient towards those aspects of my personality I don’t like.”
Common Humanity	Recognizing that imperfection and suffering are part of the shared human experience: “ I try to see my failings as part of the human condition.”
Isolation	Feeling isolated and disconnected from others versus recognizing that suffering is part of the human condition (R): “When I fail at something that’s important to me, I tend to feel alone in my failure.”
Mindfulness	Holding painful thoughts and feelings in balanced awareness rather than over-identifying with them: “When something upsets me I try to keep my emotions in balance.”
Over-Identification	Becoming consumed by one's own suffering and losing perspective (R): “When I’m feeling down I tend to obsess and fixate on everything that’s wrong.”
Posttraumatic Growth Inventory (PTGI)	
Improved Relationships	Positive changes in relationships with others: "I better accept needing others.”
New Possibilities	Seeing new opportunities and possibilities in life: “I have developed new interests.”
Personal Strength	Increased personal strength and resilience: "I have a greater feeling of self-reliance.”
Spiritual Growth	Deepening of spiritual or existential beliefs: “I have a better understanding of spiritual matters.”
Appreciation for Life	Greater appreciation for life and its experiences: “I can better appreciate each day.”
Perceived Stress Scale (PSS)	“In the last month, how often have you been able to control irritations in your life?” “In the last month, how often have you felt difficulties were piling up so high that you could not overcome them?”
Psychological Well-Being (PWB)	
Self-Acceptance	Positive attitudes toward oneself, acceptance of past life events, and a positive view of oneself: "I like most aspects of my personality"
Positive Relations with Others	Satisfying and supportive relationships with others (R): “Maintaining close relationships has been difficult and frustrating for me.”
Autonomy	Independence, self-determination, and the ability to resist social pressures (R): “I tend to be influenced by people with strong opinions.”
Environmental Mastery	Competence in managing and adapting to the environment, including being able to choose or create suitable environments: “In general, I feel I am in charge of the situation in which I live.”
Purpose in Life	Having a sense of meaning and purpose in life, feeling that one's life is meaningful and worthwhile: “Some people wander aimlessly through life, but I am not one of them.”
Personal Growth	A sense of continued development and personal evolution, striving to realize one's potential: “I gave up trying to make big improvements or changes in my life a long time ago.”

**Table 5:** Pre and Post-Test Means

Measure	Pre-Test M (SD)	Post-Test M (SD)	*t*	*p*	*d*
FFMQ	135.5 (19.7)	142.86 (22.81)	14	0.05*	0.35
Observing	28.43 (5.96)	30.36 (5.91)	14	< 0.05*	0.33
Describing	29.86 (5.72)	29.86 (6.12)	14	1.0	0.00
Acting with Awareness	26.57 (5.72)	27.93 (6.16)	14	0.2	0.23
Non Judging	28.76 (5.65)	31.14 (5.25)	14	< 0.05*	0.44
Non Reactivity	21.86 (4.27)	23.57 (4.56)	14	0.01**	0.39
SCS-SF	3.19 (0.39)	3.47 (0.50)	14	< 0.05*	0.62
PTGI	57.69 (21.07)	72.61 (20.34)	13	0.02*	0.72
Personal Strength	11.38 (4.14)	15.23 (3.72)	13	0.001***	0.98
New Possibilities	13.92 (5.48)	13.08 (5.17)	13	0.56	−0.16
Improved Relationships	18.54 (9.15)	25.46 (7.75)	13	0.005**	0.82
Spiritual Growth	3.23 (3.02)	4.54 (2.59)	13	0.04*	0.47
Appreciation for Life	10.62 (3.03)	11.69 (3.098)	13	0.11	0.35
PSS	18.07 (5.74)	15.21 (4.89)	14	0.06	−0.54
PWB	102.71 (12.29)	105.21 (12.31)	14	0.2	0.20
Autonomy	16.71 (4.35)	16.21 (3.84)	14	0.51	−0.12
Environmental Mastery	15.5 (3.18)	16.71 (3.24)	14	0.04*	0.38
Personal Growth	18.93 (1.71)	19.5 (2.23)	14	0.38	0.29
Positive Relations with Others	17.57 (3.35)	17.71 (3.15)	14	0.78	0.04
Purpose in Life	16.5 (2.97)	17.07 (3.65)	14	2.97	0.17
Self-Acceptance	18.14 (3.29)	18 (2.24)	14	0.84	−0.05

^*^p < 0.05; **p < 0.01; ***p < 0.001

#### Mindfulness

Participants exhibited a modest but non-significant increase in overall mindfulness. The Five Facet Mindfulness Questionnaire (FFMQ) showed an increase from a pre-test mean of 135.5 (SD = 19.7) to a post-test mean of 142.86 (SD = 22.81), t(14) = 1.77, *p* = 0.10, indicating that the mothers did experience positive changes in their general mindfulness. A significant increase was observed in the "Observing" sub-scale, with scores rising from pre-test (M = 28.43, SD = 5.96) to post-test (M = 30.36, SD = 5.91), t(13) = 2.41, *p* < 0.05*, reflecting improved awareness of sensory experiences. Similarly, the "Non-Judging" sub-scale showed a significant increase, with scores increasing from pre-test (M = 28.76, SD = 5.65) to post-test (M = 31.14, SD = 5.25), t(13) = 2.38, *p* < 0.05*, suggesting reduced self-criticism among the mothers. Participants also demonstrated a significant increase in the "Non-Reactivity" sub-scale, with scores improving from pre-test (M = 21.86, SD = 4.27) to post-test (M = 23.57, SD = 4.56), t(13) = 3.19, *p* = 0.01**, indicating an enhanced ability to remain unaffected by internal experiences. However, no significant changes were observed in the "Describing" sub-scale (pre-test M = 29.86, SD = 5.72; post-test M = 29.86, SD = 6.12), t(13) = 0, *p* = 1.00, or the "Acting with Awareness" sub-scale (pre-test M = 26.57, SD = 5.72; post-test M = 27.93, SD = 6.16), t(13) = 1.36, *p* = 0.20, suggesting that the mothers did not show substantial change in these specific aspects of mindfulness.

#### Self-Compassion

The Self-Compassion Scale-Short Form (SCS-SF) indicated an increase in self-compassion from a pre-test mean of 3.19 (SD = 0.41) to a post-test mean of 3.47 (SD = 0.50). This change was significant, t(13) = −2.52, *p* = 0.0256, suggesting that the participants' self-compassion increased from the start of the program to the end of the program. The.

#### Post-Traumatic Growth

**Fig. 2:**
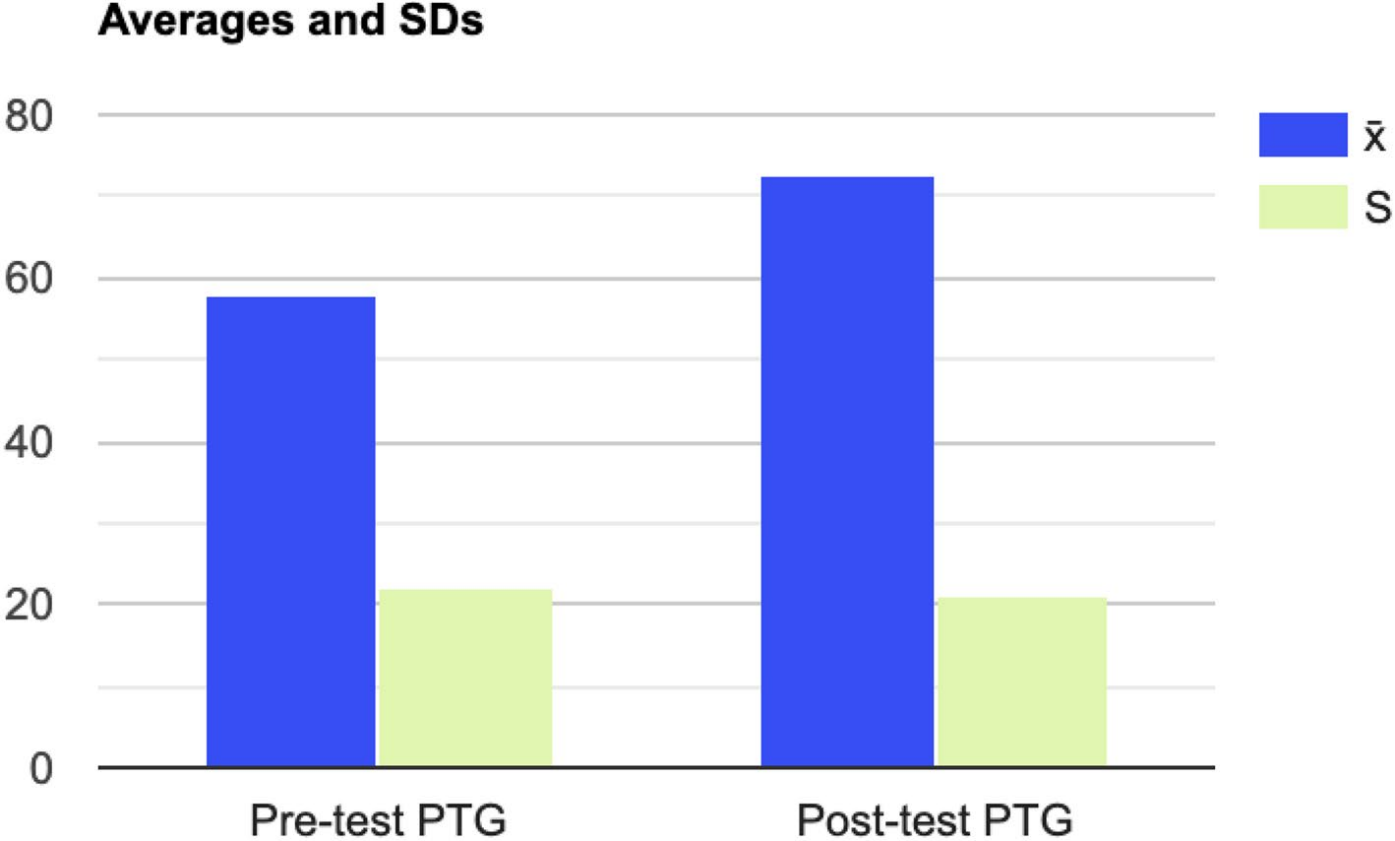
Differences in Post Traumatic Growth Inventory (PTGI) Scores

**Fig. 3:**
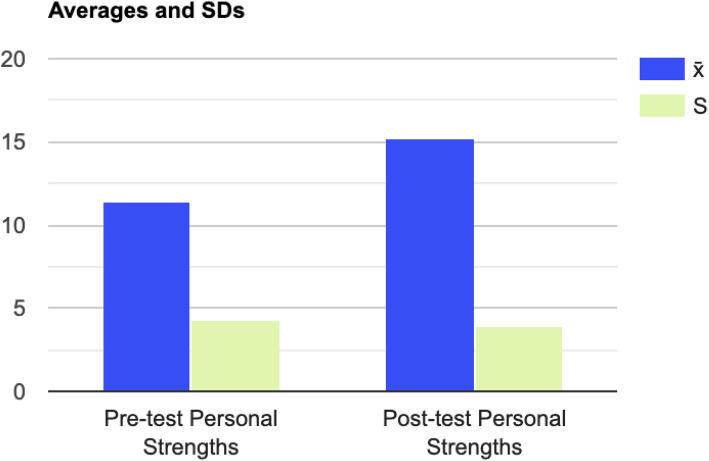
Differences in PTGI Personal Strengths Subscale Scores

**Fig. 4:**
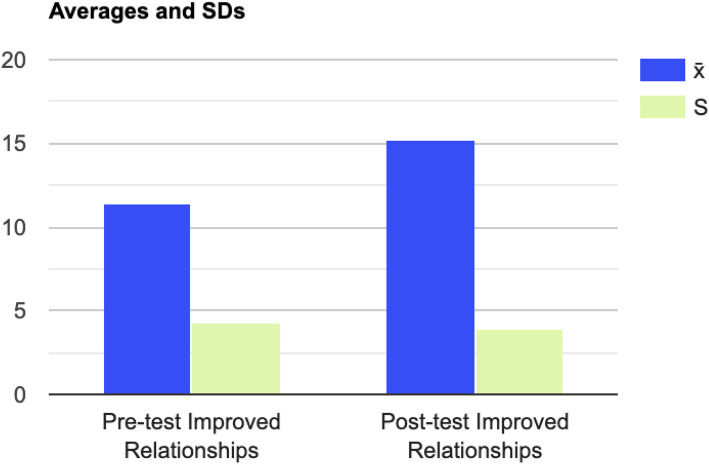
Differences in PTGI Improved Relationships Subscale Scores

Analysis of the Post-Traumatic Growth Inventory (PTGI) revealed significant changes in several areas. Overall post-traumatic growth scores increased substantially from a pre-test mean of 57.69 (SD = 21.07) to a post-test mean of 72.61 (SD = 20.34), t(12) = 2.54, *p* = 0.02*, indicating a meaningful enhancement in growth in the mothers following their experiences (see Fig. 2). The “Personal Strength” sub-scale showed a notable improvement, with scores rising from a pre-test mean of 11.38 (SD = 4.14) to a post-test mean of 15.23 (SD = 3.72), t(12) = 4.67, *p* = 0.001***, reflecting a significant increase in perceived personal strength (see Fig. 3). Enhancement was also observed in the “Improved Relationships” sub-scale, with significant changes from a pre-test mean of 18.54 (SD = 9.15) to a post-test mean of 25.46 (SD = 7.75), t(12) = 3.09, *p* = 0.005**, indicating better quality of relationships (see Fig. 4). Additionally, the “Spiritual Growth” sub-scale showed significant growth, with scores improving from a pre-test mean of 3.23 (SD = 3.02) to a post-test mean of 4.54 (SD = 2.59), t(12) = 2.23, *p* = 0.04*. In contrast, no significant changes were observed in the “New Possibilities” sub-scale (pre-test M = 13.92, SD = 5.48; post-test M = 13.08, SD = 5.17), t(12) = 0.62, *p* = 0.56, or the “Appreciation for Life” sub-scale (pre-test M = 10.62, SD = 3.03; post-test M = 11.69, SD = 3.10), t(12) = 1.68, *p* = 0.11, suggesting mothers experienced no significant shift in these areas.

#### Perceived Stress

Participants reported a non-significant decrease in perceived stress levels. Scores on the Perceived Stress Scale (PSS) changed from a pre-test mean of 18.07 (SD = 5.74) to a post-test mean of 15.21 (SD = 4.89), with a t-value of 1.98 and a p-value of 0.06. This indicates a potential reduction in stress levels among the mothers; however, the change did not reach statistical significance.

#### Psychological Well-Being

The Psychological Well-Being Scale (PWB) demonstrated a slight, non-significant increase in overall psychological well-being, with scores improving from a pre-test mean of 102.71 (SD = 12.29) to a post-test mean of 105.21 (SD = 12.31), t(13) = 1.29, *p* = 0.20, indicating limited change in participants’ overall well-being. However, significant improvement was observed in the “Environmental Mastery” sub-scale, where scores increased from a pre-test mean of 15.50 (SD = 3.18) to a post-test mean of 16.71 (SD = 3.24), t(13) = 2.21, *p* = 0.04, suggesting mothers experienced enhanced control over their environment. In contrast, no significant changes were found in the “Autonomy” dimension (pre-test M = 16.71, SD = 4.35; post-test M = 16.21, SD = 3.84), t(13) = 0.70, *p* = 0.51; the “Personal Growth” dimension (pre-test M = 18.93, SD = 1.71; post-test M = 19.50, SD = 2.23), t(13) = 0.95, *p* = 0.38; the “Positive Relations with Others” dimension (pre-test M = 17.57, SD = 3.35; post-test M = 17.71, SD = 3.15), t(13) = 0.21, *p* = 0.78; the “Purpose in Life” dimension (pre-test M = 16.50, SD = 2.97; post-test M = 17.07, SD = 3.65), t(13) = 2.97, *p* = 0.10; and the “Self-Acceptance” dimension (pre-test M = 18.14, SD = 3.29; post-test M = 18.00, SD = 2.24), t(13) = 0.19, *p* = 0.84. This indicates that these specific aspects of psychological well-being remained relatively stable throughout the duration of the program.

#### Power Analysis

A post-hoc power analysis was conducted to evaluate the adequacy of the sample size in detecting significant effects in this study. Given the small sample size (*N* = 14), the power for detecting small to medium effect sizes (Cohen’s d = 0.35) was approximately 0.80, indicating sufficient power to detect moderate effects. The following psychological measurement findings, such as self-compassion, post-traumatic growth, and environmental mastery**,** passed the power threshold, showing significant improvements with sufficient power (above 0.80) to support the findings. However, the limited sample size may have reduced the likelihood of detecting smaller effects in other measures, and would require future studies with larger, adequately powered samples to confirm these results and provide more robust conclusions.

### Qualitative Results

The qualitative analysis provided additional insights into participants' experiences with the program. These findings underscore the multifaceted nature of maternal experiences, revealing the complex interplay of challenges and adaptations that characterize the transition to motherhood. The results reveal six common themes among the mothers in the program, all of whom were navigating the transition to motherhood. Participants were assigned identification numbers for the qualitative analysis, and all supporting quotations are attributed to them using these corresponding identifiers.

#### Transition Challenges

##### Physical Changes

Participants reported significant physical changes, highlighting discomfort and altered bodily perception. For example, one respondent noted, “Physical changes- my body feels different, my pelvic floor still feels 'off', my teeth and hair feel like they have aged” (#8). Another mother described the physical toll during pregnancy and postpartum: “I had horrible morning sickness, throwing up multiple times a day through 16 weeks with both pregnancies. The third trimester was also through the summer with both kids and was uncomfortable. Finally, I was working through very stressful situations with my first” (#9).

##### Shifts in Identity

Participants reported a loss of their previous sense of self and faced challenges integrating their new maternal identity. One mother shared, “Lost my previous identity and my child constituted a big part of my new identity… but slowly regaining my own identity” (#10). Other respondents said, “My body, my identity, how I spend my time, how I prioritize things, how I think about things. Everything has changed!” (#19); and “My life is intertwined with my daughter’s life. As her full-time caregiver, it is a 24/7 experience." and that "there is much less time for myself in every aspect." (#7). This highlights the struggle to reconcile pre-motherhood identities with new maternal roles.

##### Emotional Upheaval

Mothers experienced a blend of intensified love and heightened irritation. For example, one mother stated, “I am cracked open, I feel more deeply, I feel more '2 things are true' like I have more love and patience, but also am quicker to irritation” (#2). Emotional adjustment involved coping with new stressors and navigating complex feelings related to motherhood.

##### Evolving Relationships

Many participants reported developing closer bonds with other mothers while feeling distanced from friends without children, reflecting the evolving social dynamics and relationship adjustments during the transition to motherhood. One mother noted, “Navigating partnership with stress of a newborn… not meeting my unrealistic expectations of myself” (#13), while another observed, “I've gotten closer to other moms and further apart from friends with no kids” (#6).

##### Lifestyle Adjustments

Participants highlighted significant changes in their social lives and daily routines, underscoring the complexities of integrating new responsibilities into their existing lives. Balancing work, family, and social commitments became a new challenge. One respondent mentioned, “Balancing work, family time, social calendar… Becoming a sole caregiver for another person” (#11).

##### Career Pivots

Professional adjustments were also notable, with some mothers experiencing changes in job locations or new roles, by choice or by circumstance, which added to the overall stress. For instance, a mother shared, “Job, no longer in NYC, I have a private practice in NYC I am commuting to… Honestly everything has changed” (#4). 

#### Developmental Distress

##### Exhaustion

Participants reported varying levels of difficulty, with the majority finding pregnancy challenging (Easy: *n* = 4, Moderate: *n* = 7, Difficult: *n* = 9) and motherhood even more so (Easy: *n* = 1, Moderate: *n* = 6, Difficult: *n* = 10), with many participants reporting intense physical and emotional exhaustion. One mother captured this sentiment, saying, “Motherhood is the most challenging, emotionally and physically exhausting experience I have ever had” (#7). Another noted, “I had PP mood disorder/adjustment disorder; breastfeeding has been so much more draining mentally and physically than I could’ve imagined” (#2). These experiences highlight the profound toll of childbirth and newborn care on mothers’ well-being.

##### Isolation 

Feelings of isolation were prevalent, often exacerbated by geographic relocations and insufficient support. One mother described her situation, saying, “Physically exhausting, isolating, too. Since I moved to a small non-walkable suburb in Connecticut, from Manhattan, NYC. Physically demanding to get anything done like a doctor's appointment, as it requires so much planning and coordination in terms of my partners or other ride work schedules, etc. Emotionally isolating, no support system or loving family. Very toxic family, actually” (#20). Another shared, “Pandemic made me almost completely isolated as my husband was working in a hospital” (#4). These quotes illustrate the significant impact of isolation and lack of support on the maternal experience.

##### Mental Health Symptoms

Psychological strains, including postpartum depression and mood instability, were commonly reported. One participant shared, “My transition [to motherhood] was challenging. I was extremely depressed” (#19). The prevalence of mental health challenges underscores the need for comprehensive support during the postpartum period.

##### Relational Conflicts

Challenges in relationships were frequently mentioned, including feeling unsupported by partners and experiencing marital strain. One mother said, “Love my kids but feel torn and conflicted about everything. We also moved out of state when my first was 3 months old” (#4). Another described her experience of resentment, saying, “First born was tough with the pandemic and all the uncertainty and isolation, I also resented my husband A LOT. Being married and being a new mother is 10,000% harder than raising a baby or child” (#5). These comments reflect the relational strains and adjustments new mothers face.

##### Additional Stressors

Factors such as financial constraints and workplace inflexibility were significant stressors. The pandemic further intensified these challenges, limiting access to essential services and support. One mother shared, “Pandemic hit right when I got pregnant. I received minimal prenatal care…” (#5). Another noted, “Childcare is too expensive, workplace doesn't flex for parenting” (#9). These quotes illustrate the predictable and unpredictable external pressures that compound the difficulties of the transition to motherhood.

####  Understanding Matrescence 

##### Limited Education

Participants described a broad range of educational experiences where they acquired biomedical knowledge about pregnancy, childbirth, and early-stage parenting. These included hospital birthing classes, pelvic floor therapists, doulas, and various free parenting courses offered at public libraries. They also engaged with diverse digital media such as books, podcasts, and formal support groups. For instance, one participant highlighted, “Positive/non-pathologizing birthing books and podcasts…enormously helpful and empowering. I started listening to podcasts…years before getting pregnant, so I was well-informed before conception” (#7). This indicates that these resources were deemed not only useful but essential in preparing for the practical aspects of childbirth and early parenting and were readily available to some participants.

##### Low Literacy

Despite having some knowledge of perinatal issues, many participants reported limited or partial understanding of the concept of matrescence prior to the pilot study. Most prior awareness came through informal channels such as social media, blogs, rather than academic sources like university courses and workshops. For example, one participant noted their first encounter with the concept was through "one Instagram account somewhere, along with a blog post and newsletter" (#11). Others learned about matrescence through books and popular literature, as one mother shared, "from the books I've read since becoming a mother" (#10). This suggests that matrescence has not yet achieved the same level of mainstream recognition as other aspects of maternal health education.

#####  Initial Perceptions

Before the study, participants generally described the concept of matrescence in terms of identity changes and emotional adjustments related to becoming a mother. One participant characterized it as, “the transition from maiden to mother, the stage of life of a woman where one part of herself dies to allow another to be born” (#20). Another emphasized its ongoing, evolving nature, suggesting, “It can take several years or arguably a lifetime…” (#6) much like adolescence. This reflects an initial grasp of matrescence as a profound life transition but with limited depth or exposure to more scientific explanations such as the developmental neuroscience of the brain.

##### Analogy to Adolescence

The analogy to adolescence resonated most with participants, helping them make sense of their transition to motherhood. One mother remarked, "It was mind-blowing to learn that becoming a mother was as hard as adolescence. Everything I have been through as a new mom makes sense if I see it through that lens" (#14). Another participant, who had not yet given birth, found the analogy useful for future reference: "I haven’t given birth yet, but I think it’s helpful to think of this as a life transition, like adolescence" (#12). This developmental framework, familiar from adolescence, allowed participants to more easily understand and apply the concept of matrescence to their own experiences.

##### Novel Terminology

A key benefit for participants was gaining new language to describe their maternal experiences and better articulate the changes they were undergoing. One participant noted, "I noticed the changes that have occurred, but now I have words to describe them" (#17). Another found the access to shared language affirming, stating, "It has given me words to describe what I experienced" (#2). When asked whether this language had by extension altered their comprehension or perception of the transition to motherhood, all respondents said yes. One mother stated, "It totally changed the way I think about my life and being a mother" (#8).

##### Validation of Experience

The concept of matrescence normalized and destigmatized the challenges of early motherhood for some participants. By recognizing motherhood as a developmental process, one participant shared, “Seeing it as a normal process is making me honor it more, have more respect for it and for myself, as well as more self-compassion” (#14). Another described how it, “…validated how the experience of early motherhood can feel unmoored and difficult, especially without the evolutionary village or true elders as guides through this rite of passage” (#7). The concept of matrescence in itself was experienced as validating for participants who as a result felt more understood and respected when using it.

##### Self-Acceptance

Participants reported a greater ability to accept their current life circumstances after learning about matrescence. One mother reflected, “With my first child, I really tried to just ignore the fact that I was a mother… but now I embrace it” (#8). Another participant expressed feeling more accepting and less resistant to her situation: “I am gentler with myself now” and “feel safer in my body, having acknowledged this experience and its realities. I feel like I reached a place of acceptance and less resistance” (#20).

#### Positive Growth and Adaptation 

##### Narrative Reframing

Many participants displayed a notable shift in their weekly written narratives reflecting a shift in perspective. Initially, focused on the negative aspects or detailed memories of events rather than feelings, written stories transformed, with a greater emphasis on personal meaning-making and emotions regarding their subjective journey. One participant noted, “What struck me the most is that the first one was filled with dates and events. The second one had fewer dates and more reflection on what I was experiencing and feeling throughout the process. I realized that it’s been a very lonely process, which has greatly contributed to how hard it has been” (#14).

##### Ability to Observe

Improvements in the Observing subscale of the FFMQ were reflected in participants' qualitative feedback, highlighting their enhanced capacity for mindful self-observation. Participants reported an increased awareness of their thoughts and emotions, with one mother noting that the program helped her observe her experiences and “focus on parts of myself that I was not thinking about or was not aware of” (#17). This enhancement in mindfulness allowed participants to observe their thoughts and emotions with greater clarity, contributing to their overall sense of self-awareness.

##### Emotional Regulation

Participants showed better management of negative thoughts and self-talk, as reflected in improved Non-Reactivity scores. Qualitatively, this was evident in participants’ increased ability to manage and respond to their emotions more effectively. One participant noted feeling more balanced and less overwhelmed by negative emotions, indicating the program’s impact on their emotional regulation stating that it helped, “shift my thinking about motherhood from a place of shame that I felt any negative emotions about being a mother to a place of reassurance that all women go through various emotions, thoughts, and feelings in their motherhood journey” (#3).

##### Reduced Judgment

There was a notable improvement in Non-Judgment, as participants became more compassionate toward their own emotions. This shift was evident in participants' reflections on self-acceptance and reduced self-criticism. One participant shared, “Initially I was focused on the negative parts and feeling ashamed of these thoughts. After the workshop, I am still aware of the same things, but I am more focused on what I can do” (#3). This change was further reflected in reports of increased self-love. One mother observed, “I feel my identity shifting, which in turn helps me love myself more where I am” (#8), while another expressed, “I can feel more self-compassion for myself as a new mom because it’s not that I’m doing anything wrong that’s making this process hard; it’s just hard” (#14).

##### Growth Mindset

The enhancement in participants’ growth mindset, such as viewing challenges as opportunities for growth, was evident both quantitatively and qualitatively. Quantitative results showed an increase in PTGI scores, reflecting greater openness to personal and spiritual growth. Participants expressed a shift in their outlook as one mother shared, “It is an honor and a privilege to have become a mother, despite its challenges” (#7). Another stated that she was, “full of hope, positively charged, motivated, and excited to work on getting to a place where I am thriving in my motherhood journey” and that the program, “helped transform my mindset about being a mother in a positive light” (#3).

Some begin to see their experiences through the lense of life cycles. One mother explained, “Instead of freaking out that the leaves are falling off the trees, with no true understanding why, I learned that this is a season called autumn and many things happen like the leaves falling, air getting crisper, colors changing, colds and stuffy noses brewing… that is the best way I can put it” (#20).

##### Environmental Mastery

Participants showed quantitative improvements on the PTGI subscale of Personal Strengths and the Psychological Wellbeing subscale of Environmental Mastery, as well as in their qualitative feedback. Mothers felt more competent in managing their circumstances. One participant shared, "I feel capable, I feel proud, I feel more equipped" (#11). The program’s focus on practical tools and resilience-building contributed to this change, empowering participants to proactively address their challenges. One mother explained, “Although I may be exhausted or frustrated with the lack of support I have, I know I can do something about it by digging deep and thinking about what I really need and then meeting that need. I've already started implementing things to help fill the gaps in my support system which is helping me have a more empowered and positive experience… I am more focused on what I can do to meet my needs to feel better to get to a place where I am confident and thriving” (#3).

#### Peer Sharing & Community Support

##### Improving Relations with Others

The significant improvement in the PTGI subscale of Improved Relationships was echoed in participants’ qualitative feedback about enhanced emotional vulnerability and supportive relationships. Participants reported feeling more connected and supported in their relationships, reflecting the positive impact of the program on their social connections. One mother noted that it, “helped me gain confidence to open up more about my experience and want to share my authentic self and experience to connect with others” (#3). Many (five) of the participants also shared how the program made them feel “less alone” or helped them realize they are “not alone” in their experience.

##### Power of Peer Sharing

Participants valued the group dynamics of the educational program, reporting that peer sharing reduced feelings of isolation and increased their sense of agency and belonging. One participant remarked, “I was amazed by the vulnerability of each person and was happy to know this was a safe space for everyone to share,” and added, “It felt great to hear the similarities and differences in what each person was experiencing” (#3), underscoring the validation received by supportive peers. Another participant expressed how empowering it was to be, “part of a group of women who are talking about their experience with being a new mom” (#7). These indicate that the shared community experience played an instrumental role in alleviating mothers’ loneliness and enhancing their sense of belonging.

##### Assessing External Resources

The program facilitated a deeper understanding of available resources and support networks. Participants reflected on how to more frequently engage in help-seeking behavior. One mother noted, “For my next child, I already know what are some things/people/resources/support to put in place” (#20), illustrating planning ahead for future adversity. Another said, “It helped me understand that I have been missing connection and rituals” (#14). This proactive approach to seeking and utilizing resources underscores the program’s impact on enhancing participants' awareness and implementation of external support.

#### Perceived Acceptability, Reliability, & Benefits

##### Acceptability

Participants expressed a high level of satisfaction with the program, with an overall positive rating. The program was praised for its relevance and applicability to their lives as mothers. While feedback was generally favorable, one participant who attended two sessions reported lower satisfaction. Despite this, the majority valued the program's organization, presentation, and accessibility, particularly appreciating the flexibility of the live online format. Components such as group discussions, knowledge sharing, and meditation practices were well-received, though some found certain aspects less engaging than others. Participants were overall enthusiastic with one stating that, “I wish every new mother was provided with this important information and support” (#6). However, it is important to note the potential bias of social desirability in participants' responses, as they may have reported higher levels of satisfaction due to a desire to align with perceived program expectations, group norms, or conceal feelings of discomfort or dissatisfaction.

##### Reliability

Participants valued the program’s academic foundation and the credibility provided by its university affiliation. This association reassured them about the quality and reliability of the content. As one participant noted, “It helps that this is through a recognized university of high standing, within a respected department. If it wasn’t, the word matrescence could easily be discarded and grouped with other ideologies, perhaps perceived as crunchy or new age. The university affiliation reassures me about the quality and reliability of the information provided” (#20). Additionally, the program fostered a safe and supportive environment, cultivating a strong sense of safety and empathy among participants. One mother expressed her appreciation, saying, “I felt such a sense of safety and empathy from the facilitators and the other women in the group that I was happy to share and participate each week” (#3).

##### Benefits

The program was credited with enhancing participants’ understanding of matrescence and providing essential support. Many felt that the workshop played a significant role in their personal growth and emotional well-being, normalizing the challenges of motherhood while offering valuable tools and resources. One participant noted, “The understanding of matrescence and acknowledgment of it lifts a stigma and eases suffering through showcasing and teaching what this time period actually is, why it is hard, and what are some tools and resources that can make it better. Often, therapy and medication are barely even a band-aid. But this workshop on matrescence is actual medicine” (#20). Many wished to recommend the workshop to others, highlighting its empowering and transformative impact. As one participant shared, “Every mother should be required to take a course on Matrescence! It has totally changed the way I think about my life and being a mother” (#8).

## Discussion

The transition to motherhood is a complex and multifaceted process. Our study underscores both the labyrinthine experiences new mothers face and the benefits of a targeted educational program centered on matrescence to better orient them. Consistent with existing research, participants reported a range of challenges during pregnancy and early motherhood, including physical changes, shifts in identity, emotional exhaustion, relational dynamics, social adjustments, isolation, insufficient support, mood instability, career transitions, and extended family stressors. The impact of these challenges varied with each participant, emphasizing the individualized nature of the maternal transition. These findings reinforce the importance of identifying the stressors each mother may uniquely experience within the various domains of matrescence which may make them more vulnerable to postpartum mood and anxiety disorders.

Quantitative results revealed notable improvements in psychological constructs associated with well-being and resilience. While the overall increase in mindfulness was modest and not statistically significant, specific subscales showed significant gains. The Observing subscale improved significantly, indicating heightened awareness of sensory experiences, including thoughts and feelings. The Non-Judging subscale also demonstrated significant progress, reflecting a reduction in self-criticism and an increased capacity for non-judgmental awareness. Additionally, the Non-Reactivity subscale showed significant enhancement, suggesting improved emotional stability in the face of internal challenges. Notably, mothers in this sample scored relatively high on the FFMQ, with pre-test scores averaging 135.5 and post-test scores averaging 142.86. These scores are comparable to populations who meditate, which tend to have higher mindfulness scores compared to non-meditating populations [60, 96, 8].

Participants not only demonstrated an increase in self-compassion, with notable improvement from the beginning to the end of the program, but also began with relatively high SCS-SF scores compared to the general, non-clinical population [81]. Substantial post-traumatic growth was observed, particularly in areas of Personal Strength and Improved Relationships, with participants reporting significant gains in both. Enhancements in Spiritual Growth were also evident, indicating that mothers found greater meaning in their experiences and developed a deeper connection to their values and beliefs. Additionally, a significant increase in the Environmental Mastery subscale from the Psychological Wellbeing Scale suggested that participants gained a stronger sense of control over their surroundings and daily life challenges. These findings indicate that the program may have facilitated participants to make more meaning of their maternal experiences that lended a sense of personal growth and self-mastery. Notably, these positive changes corresponded with participants’ reassessment of their values and beliefs through learning about matrescence, allowing them to approach challenges with greater self-compassion, acceptance, optimism, and confidence. This transformation highlights the program’s potential to build resilience and serve as a protective measure against common cognitive vulnerabilities in motherhood, such as perfectionism, self-criticism, anxiety, and guilt. However, the lack of significant changes in perceived stress and overall psychological well-being suggests that broader well-being may require more intensive or long-term interventions.

Qualitative insights from the study captured the depth and complexity of emotions, as well as the developmental distress commonly encountered by mothers during this period. Initially, many participants had a limited understanding of matrescence, but the program deepened their appreciation for the concept, validating and normalizing their experiences when framed as a developmental process. This reframing allowed participants to approach motherhood with greater confidence. The study also emphasized the importance of knowledge, coping skills, and peer support in reducing isolation and empowering participants through improved health literacy. The vast majority of mothers reported that learning about the concept of matrescence was highly valuable and most considered the self-reflective practices helpful as well. The subject that participants seemed least interested in or affected by was the didactic portions on the psychology of transformation. Reflection and shared processing with others, along with support networks, were highly valued, though some participants expressed lower satisfaction due to limited attendance. Many regretted not having access to such support earlier, highlighting the need for more widespread and earlier maternal education throughout the lifespan. Despite significant stressors such as exhaustion and isolation, participants demonstrated resilience and a newfound openness, suggesting that a strengths-based approach to the transition to motherhood can prime mothers for growth and adaptation.

Our findings also revealed a significant gap between the biomedical resources available to mothers and the need for comprehensive psychological, social, and developmental education. Despite their privilege and higher education, participants entered motherhood with insufficient knowledge about its complexities, challenging the notion that motherhood is purely instinctual. Just as adolescent sexual health education has evolved to incorporate psychosocial elements, it is essential to introduce similar approaches to maternal health that extend beyond anatomy and physiology. By integrating tools from psychological science and developing more matrescence-informed educational programs, new mothers may be better prepared for the lifelong emotional demands and chronic stress of parenting. This shift requires broadening existing maternal health dissemination channels to include emotional, psychological, and developmental preparation, alongside traditional childbirth education. Additionally, professionals and paraprofessionals must receive specialized training to deliver this education competently, affordably, and ethically. Through the power of public education, mothers from all communities and capabilities, not just the privileged few, can navigate the challenges of motherhood with greater confidence and resilience.

The findings from this pilot study contribute to the growing body of literature on matrescence by providing a nuanced understanding of how mothers experience and navigate this developmental transition. Through direct quotes from participants and the measurement of psychological constructs such as self-compassion, mindfulness, and personal growth, the study emphasizes the need for a holistic approach to maternal health, addressing the physical, emotional, social, and psychological dimensions of motherhood. Furthermore, the study furthers our understanding of maternal resilience and suggests dissemination of early intervention programs, such as matrescence education, to support mothers during this critical life stage.

### Limitations

The findings of this study provide valuable insights into the potential benefits of the matrescence-informed maternal health education program, but several limitations should be acknowledged. First, the small sample size limits the generalizability of the results and may not fully reflect the diversity of experiences within the broader population. Dropouts from participants reduced the statistical power of the analysis and may have introduced bias. Additionally, varied attendance across sessions complicates the interpretation of the results, as inconsistent participation could have influenced the intervention's effectiveness. The absence of a control group receiving no intervention or an alternative approach also limits the ability to attribute improvements in maternal well-being solely to the matrescence-based curriculum. Future studies should include randomized controlled trials (RCTs) with adequately powered sample sizes to better understand the effects of matrescence education on mothers. Additionally, a larger observational or longitudinal study could provide further insights into the program's long-term effectiveness and broader applicability. Future research should aim to use randomized controlled trials with larger sample sizes and more consistent attendance to validate the program’s efficacy as well as long-term follow-ups to assess the lasting effects on maternal well-being or later development of depression or anxiety.

Additionally, while the study explored the potential influence of parity and time of onset of matrescence on reported experiences and well-being, no observable differences were noticed while attending sessions by the facilitators. The broad range of maternal stages—from pregnancy and early parenting—introduced variability, as each have their own unique psychological and emotional challenges. The disparity between the number of mothers who expressed interest in the program, those who enrolled, and those who ultimately completed it highlights the difficulty of recruiting this overwhelmed and hard-to-reach demographic for research. Future studies should consider grouping participants by maternal stage, parity, and age of the focus child to more accurately delineate the distinct developmental tasks of each group. Stratifying participants in future research could help isolate specific impacts and enhance understanding of these influences on maternal mental health and well-being or, alternatively, studies could blend experienced and inexperienced mothers to research the benefits of such wisdom-sharing. Additionally, the sample’s predominantly high education level and marital status may have influenced their enhanced access to resources and support networks and overall comprehension of matrescence.

To understand why mothers did not show significant improvements in overall mindfulness, perceived stress, and psychological well-being, it is important to consider the gradual and iterative nature of wellness practices. Given deeply ingrained cognitions and behaviors, sustained engagement is likely required for more substantial sustained improvements. The positive psychology theory of “Broaden and Build” supports this, suggesting that positive emotions foster resilience and psychological well-being over time [36]. Participants also reported challenges integrating various wellness practices into their daily routines. To address this, future iterations of the program could incorporate more accessible tools, such as pre-recorded meditations, to facilitate regular practice and encourage continuing engagement. Despite the small sample size of this pilot study and lack of control group, which precludes direct causation; the results are promising and suggest valuable future directions. Further research into matrescence educational programs is warranted to build on these initial findings.

## Future Directions and Conclusions

Ensuring the accuracy and credibility of information is particularly essential in maternal health education when engaging vulnerable new mothers. This pilot study’s educational program was grounded in established theory and employed methodologically rigorous approaches, including the use of validated psychological tools to assess outcomes. These instruments provided reliable measures of the program’s impact on participants’ well-being and resilience. Participants reported a strong sense of trust and safety in the program, which they attributed to its structured design and affiliation with a respected graduate school with a specialized reproductive health psychology faculty and students, ensuring adherence to high academic and professional standards. This feedback highlights the importance of delivering maternal education that is both accessible and vetted by credible institutions. To responsibly apply theoretical concepts like matrescence to maternal healthcare in the real world, it is essential to develop best practices and industry standards. This will ensure that implementation is both ethical and effective, minimizing potential harm to participants. Future research should focus on creating standardized education or certification for matrescence care, investigating the role of mentorship and supervision in maintaining high-quality practices, and exploring how to foster ongoing professional development in the field.

Research efforts should also focus on reducing barriers to matrescence education, particularly for underserved communities, by developing outreach programs for low-income and marginalized groups and expanding access through community centers, healthcare facilities, and online platforms. Integrating community values into care models and building interdisciplinary networks are essential to prevent vulnerable mothers from falling through gaps in healthcare systems. The consideration of the language of “mother” should also be addressed as other terminology may be more inclusive for a wider population. Additionally, leveraging digital platforms can enhance accessibility and convenience, aligning with contemporary digital culture. Addressing these areas will help ensure that all mothers have the resources and support they need to thrive during this transformative period. By addressing these areas through evidence-based education and interdisciplinary collaboration, we can create a more inclusive and supportive maternal care landscape that benefits both mothers and their families.

## Data Availability

No datasets were generated or analysed during the current study.
